# Human Rights and the Tobacco Industry: An Unsuitable Alliance

**DOI:** 10.15171/ijhpm.2018.03

**Published:** 2018-01-23

**Authors:** Brigit Toebes

**Affiliations:** Global Health Law Groningen Research Centre, Department of Transboundary Legal Studies, Faculty of Law, University of Groningen, Groningen, The Netherlands.

## Dear Editor,

The United Nations (UN), non-governmental organizations (NGOs) and human rights experts have increasingly insisted on the human rights responsibilities of non-state actors including private businesses and multinational corporations. Given the power and influence that they exert over our wellbeing and over our health, many stakeholders no longer find it acceptable that private businesses can simply ignore human rights.^1^ More specifically, many stakeholders have insisted on the incompatibility of the tobacco business with human rights. For example, the UN Global Compact decided in 2017 to officially exclude tobacco companies from participating in the initiative.^2^ In that same year, the Danish Institute for Human Rights ended its engagement with Philip Morris International.^3^

Unlike governments, private businesses including the tobacco industry are not formal parties to the human rights treaties, as result of which they do not carry direct legal obligations under these documents. However, over the past decennia the approach has changed. It is now broadly accepted that private actors, even though they are not signatories to the human rights treaties, carry responsibilities to ‘respect’ human rights. The former rapporteur for Business and Human Rights, John Ruggie, stipulated this responsibility in his authoritative 2011 Guiding Principles on Business and Human Rights.^4^ In a nutshell, the responsibility to respect human rights means ensuring that human rights and their underlying values are not brought any harm. This does not go as far as the governmental obligation to respect, protect and fulfil human rights, which also includes positive duties to regulate and to oversee the actions of non-state actors, and to ensure access to necessary services, including education and healthcare.^5^

So what does the responsibility to respect entail for the tobacco industry?^6^ Importantly, it includes a responsibility not to harm human rights through the products that it brings on the market. This means that human rights, including the right to life and the right to health, need to be respected with the production, marketing and sales of its products. For the tobacco industry, this makes a very clear case: by producing, marketing and selling a product that is deadly by design, the tobacco industry flagrantly violates this human rights responsibility. As a consequence, producing, marketing and selling tobacco is fundamentally incompatible with human rights. Human rights responsibilities thus force the tobacco industry to go out of business.

Whilst working towards this goal, the tobacco industry should respect human rights throughout its value chain, starting with respecting the working conditions of its employees, strengthening the information provision on the toxic nature of tobacco, as well as reducing the harmfulness of its products.^7^

## Acknowledgements

The author receives funding from the Dutch Cancer Society (KWF), Amsterdam, the Netherlands for legal research in tobacco control. This organization did not play a role in the design of the study, nor in writing the manuscript.

## Ethical issues

Not applicable.

## Competing interests

Author declares that she has no competing interests.

## Author’s contribution

BT is the single author of the paper.
